# VIEWS FROM THE MASTERS: Pocket ultrasound devices: time to discard the stethoscope?

**DOI:** 10.1530/ERP-14-0062

**Published:** 2014-08-28

**Authors:** Sanjiv Kaul

**Affiliations:** Knight Cardiovascular Institute, UHN-62, Oregon Health and Science University, 3181 SW Sam Jackson Park Road, Portland, Oregon, 97239, USA

The stethoscope is an air-filled tube that conducts sound. It replaced the direct placement of the ear on the chest more than 200 years ago. We now have two-dimensional ultrasound allowing us to visualize cardiac structure and function in real time. The addition of Doppler has also facilitated the assessment of blood flow through the cardiac chambers and great vessels. Over the past decade, smaller and smaller ultrasound devices have been designed, such that we now have devices that fit in the pocket of a white coat. These devices have been tested against standard high-end echocardiography systems and found to be comparable in their ability to detect important cardiac abnormalities. It is also possible to train novices in the use of these devices to exclude major cardiac abnormalities. Many medical schools now use these devices in order to train students in point-of-care ultrasound for many medical and surgical conditions, including those that affect the heart. The question is whether these devices will gradually replace the stethoscope for point-of-care evaluation of patients.

Several studies carried out over the past three decades have demonstrated the major limitations of the stethoscope. Only one-quarter to one-half of the important cardiac abnormalities can be identified by medical students and residents (1, 2). Observer agreement on the presence of the third or fourth heart sounds is from poor to modest at best (3). Training with simulation (such as Harvey) did not improve the ability of students to detect cardiac abnormalities (2).

Several studies have compared small ultrasound devices with physical examination using standard echocardiography as the reference (4, 5, 6, 7). In all studies, the ultrasound devices were found to be significantly superior to physical examination for detecting cardiac abnormalities. The time taken for the performance of the ultrasound examination was only a few minutes (2, 3) more than the cardiac physical examination (5, 6). Interestingly, if no abnormality was found on ultrasound examination, physicians were less likely to order additional tests than when no abnormality was found on physical examination (5, 6), suggesting a greater confidence in making the diagnosis with the ultrasound device. Ordering fewer tests can potentially reduce the total cost of care. In the USA, it is estimated that unnecessary tests constitute one-sixth of the total health care cost.

Contemporary cardiologists are all trained in echocardiography and can therefore use pocket ultrasound without any difficulty. The question is whether other physicians can be trained in the use of these devices. Once again, there are several studies indicating that medical students, residents, and internists can be easily trained in the use of these devices in a short period of time (7, 8, 9). Once trained in the use of these devices, medical students and residents perform better than experienced cardiologists relying on the stethoscope alone (10).

What are the obstacles for the use of pocket ultrasound? First of all is physician inertia. Second is the reluctance to trade-in an inaccurate and unreliable tool with which physicians are comfortable for a more accurate and reliable tool with which they are not, and which may require some additional training and learning. In a fee-for-service environment, there is no incentive to use these devices. However, as we move to capitated systems of care, the cost of additional, unnecessary testing could become prohibitive, thus forcing us to use pocket ultrasound to make sure that we order tests only in patients in whom cardiac imaging at rest does not explain the symptoms.

In summary, pocket ultrasound devices provide high-quality diagnostic images of the heart in real time. These devices are relatively easy to use and far more accurate than the stethoscope. Their use can potentially decrease additional expensive tests. These devices bring us into the present and propel us into the future. It is time to discard the inaccurate albeit iconic stethoscope and join the rest of mankind in the technology revolution!

## References

[1] Mangione S Nieman LZ Cardiac auscultatory skills of internal medicine and family practice trainees Journal of the American Medical Association 278 1997 717–722. 10.1001/jama.1997.03550090041030 9286830

[2] Oddone EZ Waugh RA Samsa G Corey R Feussner JR Teaching cardiovascular examination skills: results from a randomized controlled trial American Journal of Medicine 95 1993 389–396. 10.1016/0002-9343(93)90308-C 8213871

[3] Lok CE Morgan CD The accuracy and interobserver agreement in detecting the ‘gallop sounds’ by cardiac auscultation Chest 114 1998 1283–1288. 10.1378/chest.114.5.1283 9824002

[4] Spencer KT Anderson AS Bhargava A Bales AC Sorrentino M Furlong K Lang RM Physician-performed point-of-care echocardiography using a laptop platform compared with physical examination in the cardiovascular patient Journal of the American College of Cardiology 37 2001 2013–2018. 10.1016/S0735-1097(01)01288-8 11419879

[5] (2014). Hand-held ultrasound versus physical examination in patients referred for transthoracic echocardiography for a suspected cardiac condition. JACC. Cardiovascular Imaging.

[6] Greaves K Jeetley P Hickman M Dwivedi G Sabharwal N Lim T Janardhanan R Senior R The use of hand-carried ultrasound in the hospital setting – a cost-effective analysis Journal of the American Society of Echocardiography 18 2005 620–625. 10.1016/j.echo.2004.09.015 15947762

[7] Panoulas VF Daigeler AL Malaweera AS Lota AS Baskaran D Rahman S Nihoyannopoulos P Pocket-size hand-held cardiac ultrasound as an adjunct to clinical examination in the hands of medical students and junior doctors European Heart Journal Cardiovascular Imaging 4 2013 323–330. 10.1093/ehjci/jes140 22833550

[8] Alexander JH Peterson ED Chen AY Harding TM Adams DB Kisslo JA Jr Feasibility of point-of-care echocardiography by internal medicine house staff American Heart Journal 147 2004 476–481. 10.1016/j.ahj.2003.10.010 14999197

[9] Hellmann DB Whiting-O'Keefe Q Shapiro EP Martin LD Martire C Ziegelstein RC The rate at which residents learn to use hand-held echocardiography at the bedside American Journal of Medicine 118 2005 1010–1018. 10.1016/j.amjmed.2005.05.030 16164888

[10] Kobal SL Trento L Baharami S Tolstrup K Naqvi TZ Cercek B Neuman Y Mirocha J Kar S Forrester JS Comparison of effectiveness of hand-carried ultrasound to bedside cardiovascular physical examination American Journal of Cardiology 96 2005 1002–1006. 10.1016/j.amjcard.2005.05.060 16188532

